# An urgent health problem of indoor air pollution: results from a 15-years carbon monoxide poisoning observed study in Jinan City

**DOI:** 10.1038/s41598-023-28683-0

**Published:** 2023-01-28

**Authors:** Mingjun Li, Bing Shan, Xiumiao Peng, Huiyun Chang, Liangliang Cui

**Affiliations:** 1 Department of Research and Education, Jinan Municipal Center for Disease Control and Prevention, Jinan Municipal Center for Disease Control and Prevention Affiliated to Shandong University, 2 Weiliu Road, Huaiyin District, 250021 Jinan, China; 2 Department of Environmental Health, Jinan Municipal Center for Disease Control and Prevention, Jinan Municipal Center for Disease Control and Prevention Affiliated to Shandong University, 2 Weiliu Road, Huaiyin District, Jinan, 250021 China

**Keywords:** Environmental social sciences, Risk factors

## Abstract

Carbon monoxide (CO) poisoning is a public health concern in developing countries especially in China with a high disease burden. We aimed to focus on non-occupational CO poisoning caused by household coal heating secular trends based on registry data in Jinan, China, and we aim to provide further evidence and suggestions for public health policy. We analyzed the occurrence and development trend and assess the spatial–temporal epidemiological characteristics of non-occupational CO poisoning caused by household coal heating in Jinan between 2007 and 2021. Among total of 6588 CO poisoning, 5616 cases (85.25%) and 180 deaths caused by household coal heating was identified during study period. The cumulative incidence rate was 5.78 per 100,000 person-years and the mortality rate was 0.19 per 100,000 person-years. The incidence in urban areas (6.55 per 100,000 person-years) was higher than rural areas (5.04 per 100,000 person-years), and there was a statistical difference between urban and rural (P < 0.001) (P < 0.001). The poisoning time point mainly occurs in the sleep stage. In Jinan, socioeconomic status, accessibility to health services and rural status are determinants for CO poisoning incidence and mortality. Implementation of urban and rural central heating renovation is an effective way to further reduce the disease burden of CO poisoning in the future.

## Introduction

Indoor air pollution sources from outdoor diffusion and indoor human activities have become the leading cause of the disease burden^1^. Usually, indoor air has the same pollutants as outdoor and could cause the same disease effects, such as particulate matter (PM), sulfur dioxide (SO_2_), nitrogen dioxide (NO_2_), and carbon monoxide (CO). However, the health effect and the emergency poisoning event caused by ambient CO were rare, while this usually occurred in an indoor environment with limited space and high concentration has become an urgent public health problem worldwide^2^.

CO poisoning begins with inhaling relatively high CO concentration^3,4^, evenly a small amount of CO dissolved in blood could replace the binding of oxygen^5^, resulting in a series of diseases and even death. A Global epidemiological study of CO poisoning showed the incidence and mortality of CO poisoning were 137 cases and 4.6 deaths per million in 2017, which made a 5.1 percent increase in incidence and a 100 percent decrease in mortality compared with 1992^6^. It’s worth noting that China reported an incidence of 56.3 cases per million and mortality of 1.93 deaths per million in 2017, which was twice lower than the global level^7^.

China is one of the challenges countries faced with CO poisoning problems. It is located in the east of Asia and on the west coast of the Pacific Ocean, with a land area of 9.6 million km^2^, a large north–south span, and a complex climate^3,8^. According to the zero-temperature isotherm line of winter in China, the south region was temperature classified by 0 °C and above, while the north was below 0 °C. Therefore, people in the northern often burn coal for heating in winter^9,10^. The CO poisoning features in the distinct south-north in China vary on the living environment, household heating and cooking, and poor ventilation^9,11^. Differently, deliberate exposure to gas in suicide is the dominant cause of CO poisoning in some developed countries^12^.

Target local actions and control measures for CO poisoning prevention addressed further concerns. A total of 27 855 non-occupational CO poisoning cases were reported between 2017 and 2021 in China, present decrease in mortality^13^. It indicated that with the improvement of medical technology and the expansion of services supply covered in recent years, CO poisoning cases could receive better treatment leading to a decreasing death, but people's awareness of CO poisoning prevention was still weak.

In the recent 5 years, 90% of the CO poisoning cases of China occurred in the northern region, and Shandong Province got the first with nearly half cases reported^13,14^. CO poisoning clustered consistently with to the high density of the population. In the study, we selected Jinan city with a 10 million population, the capital of Shandong province, to conduct a 15-years CO poisoning event observed study and describe the long-term spatiotemporal characteristics and progress, and we aim to provide further evidence and suggestions for public health policy.

## Methods

We firstly counted the number of indoor CO poisoning events and related cases, and then estimated the incidence and mortality rate in Jinan from 2007 to 2021 overall and by year individually. Further, we described the spatial and temporal characteristics of CO poisoning. In terms of space, we compared the difference between urban and rural districts by drawing map. In terms of time, we respectively described the prone time of CO poisoning in yearly, monthly, and hourly distribution. Finally, we explored the individual risk to CO exposure-disease-death relationship and performed trend fitting. The method steps and details as following description:

### Study participants

The study was approved by the Ethics Committees of the School of Public Health, Shandong University (the identification code was LL20220922) and informed consent was obtained from all participants and/or their legal guardians. The study complied with the Declaration of Helsinki.

### Background information of Jinan

Jinan is located at 36.40° N latitude and 110.00° E longitude and comprises a total of 8,177.21 km^2^. It is divided into ten administrative districts including five urban area districts of Lixia, Shizhong, Huaiyin, Tianqiao, Licheng and five rural area districts of Changqing, Zhangqiu, Jiyang, Pingyin and Shanghe^15^. It has a temperate monsoon climate with unevenly distributed seasons and the winter time lasting for nearly 150 days (from November to the next March)^16^. The minimum temperature in winter is often below −10 °C and the extreme weather such as cold waves, snowstorm occasionally occurred in January and December. According to *Jinan Urban Central Heating Management Regulations*^17^, urban region were provided central-heating services from March 15 to November 15 (121 days every year), while in rural and urban–rural joint areas, coal heating is often used due to substandard facilities, which greatly increases the risk of CO poisoning.

### Data collecting

Daily meteorological data during 2007 to 2021 were supplied by China Meteorological Data Sharing Service System network^18^, including daily mean temperature, daily maximum temperature and daily minimum temperature.

The daily non-occupational CO poisoning event profiles in Jinan from 2007 to 2021 were obtained from *China Public Health Emergency Information System*^19,20^. The system running based on the 91 notifiable reporting hospitals and integrated outbreak/event profiles on infectious disease, food-borne disease, water-borne disease, occupational poisoning, environmental emergencies (indoor and outdoor air pollution), and other unknown public health events. Non-occupational CO poisoning event was involved in the indoor environmental emergencies. We collected CO poisoning cases viables include illness onset date (hospital visiting time), poisoning location and place, poisoning reason, CO exposure population, and poisoning severity.

The main causes of non-occupational CO poisoning in Jinan include household coal heating, gas leakage in catering, suicide and others (caused by other events). From 2007 to 2021, CO poisoning caused by household coal heating accounted for 81%, which was the main reason of CO poisoning. Therefore, spatiotemporal characteristics and incidence trend of household coal heating were analyzed in this study.

### Analysis of epidemiological characteristics

Using descriptive epidemiological methods to analyze the temporal, spatial and population characteristics of CO poisoning caused by household coal heating. Exposure rate, incidence density (cases) and mortality rate were measured using the following formula:

$$\mathrm{Exposure\, Rate }= \frac{\mathrm{Number \,of \,CO \,Exposed \,Individuals}}{\mathrm{ Annual \,Population }\,\times \,\mathrm{ Time \,}(\mathrm{years})}\,\times\, \mathrm{100,000}$$

(units: per 100,000 person-years).

$$\mathrm{Incidence \,Density }= \frac{\mathrm{Number \,of \,CO \,Poisoning\, Cases}}{\mathrm{Annual\, Population\, }\times \,\mathrm{ Time \,}(\mathrm{years})}\,\times \,\mathrm{100,000}$$

(units: per 100,000 person-years).

$$\mathrm{Mortality\, Rate }= \frac{\mathrm{Number \,of \,CO \,Poisoning \,Deaths}}{\mathrm{Annual \,Population }\,\times\, \mathrm{ Time\, }(\mathrm{years})}\,\times \,\mathrm{100,000}$$

(units: per 100,000 person-years).

### Statistical analysis

We established an Excel database and used descriptive epidemiological methods to analyze the data. The spatial analysis was performed by RStudio version 3.5.1. The statistical analyses were performed by SPSS 21.0 (SPSS Inc., Chicago, IL, USA). Chi-Square test was used to compare rates, the statistical significance level was set at 0.05.

### Ethics approval and consent to participate

This study was approved by the Ethics Committees of the School of Public Health, Shandong University (the identification code was LL20220922) and informed consent was obtained from all participants and/or their legal guardians. The study complied with the Declaration of Helsinki.

## Results

### Basic situation of Jinan

From 2007 to 2021, a total of 6021 emergencies/events and related 6588 cases were reported on the *China Public Health Emergency Information System*. Of them, 5 024 events (83.44%) were non-occupational CO poisoning. The main events of CO poisoning were coal heating (4,093, 81.47%), hot pot gas leakage (588, 11.70%), suicide (13, 0.26%) and others (336, 7%). The incidence density of household coal heating CO poisoning in Jinan family from 2007 to 2021 was 5.78 per 100,000 person-years, and the mortality rate was 0.19 per 100,000 person-years.

### Spatial analysis

#### Differences between urban and rural areas

**Figure 1:**
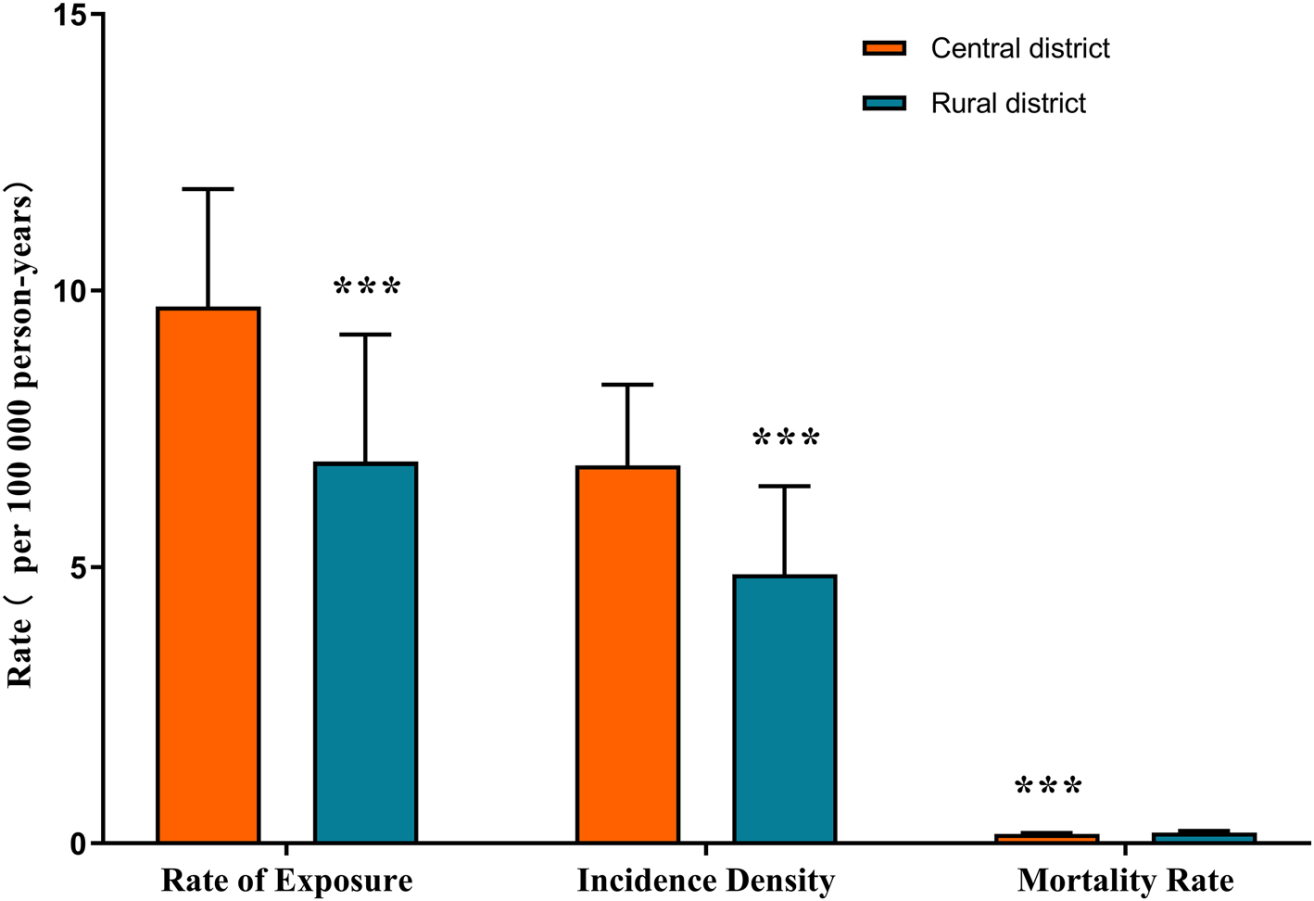
Comparison of exposure, incidence and mortality rate in central district and rural district.

**Figure 2:**
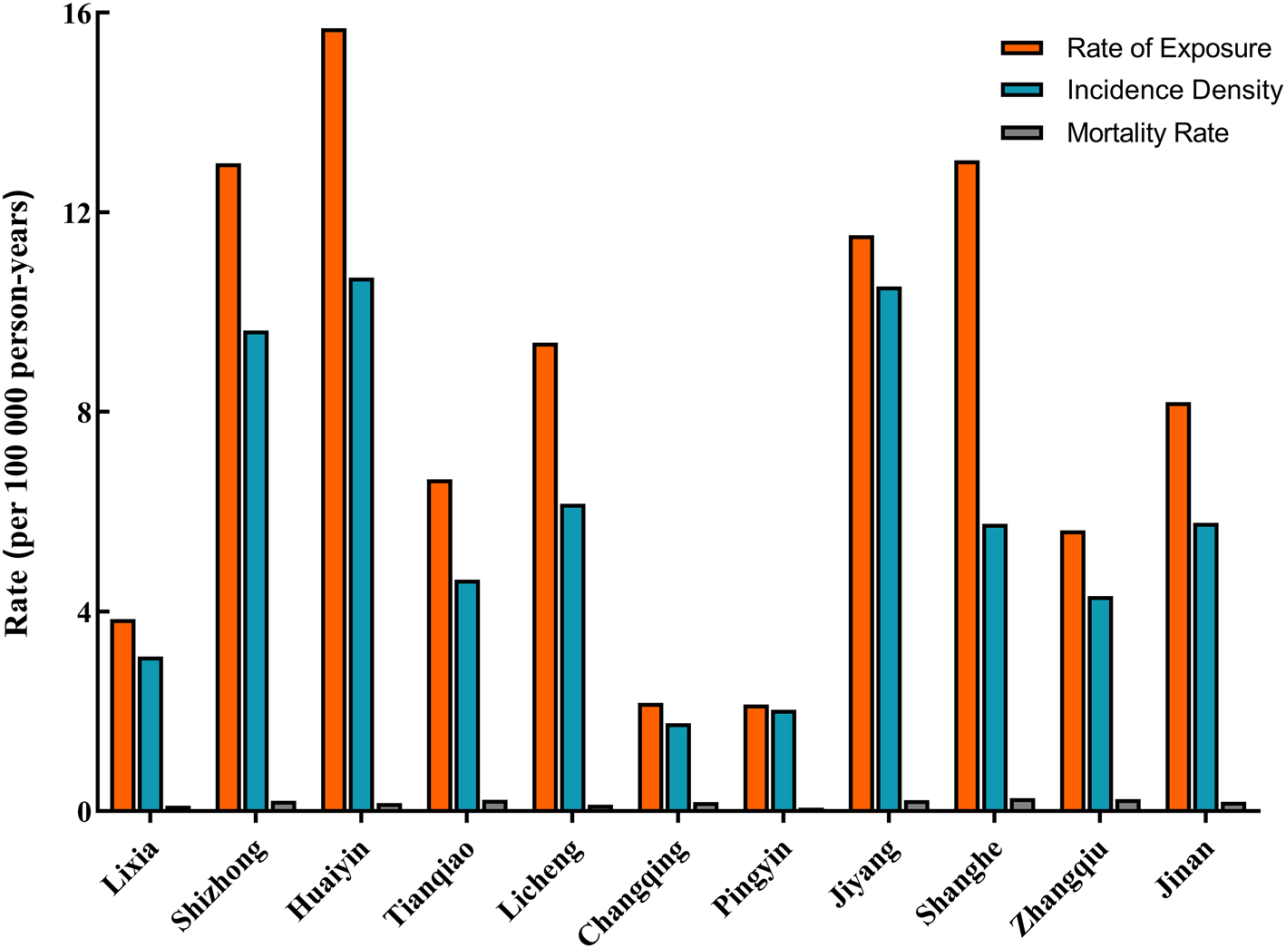
Comparison of exposure, incidence and mortality rate in all districts of Jinan.

Differences in CO poisoning in urban and rural areas were shown in Figs. 1 and 2. CO poisoning incidents occurred in all districts of Jinan, even the CO exposure, incidence rate, and mortality rates in urban areas are higher than rural areas (*P* < 0.001) (Fig. 1). Among all districts in Jinan, Pingyin district had the lowest exposure (2.14 per 100,000 person-years), incidence (2.03 per 100,000 person-years) and mortality rates (0.07 per 100,000 person-years), Huaiyin district had the highest exposure (15.69 per 100,000 person-years) and incidence rates (10.69 per 100,000 person-years), and Shanghe district had the highest mortality rate (0.26 per 100,000 person-years) (Fig. 2).

#### Spatial distribution during 2007–2021

**Figure 3:**
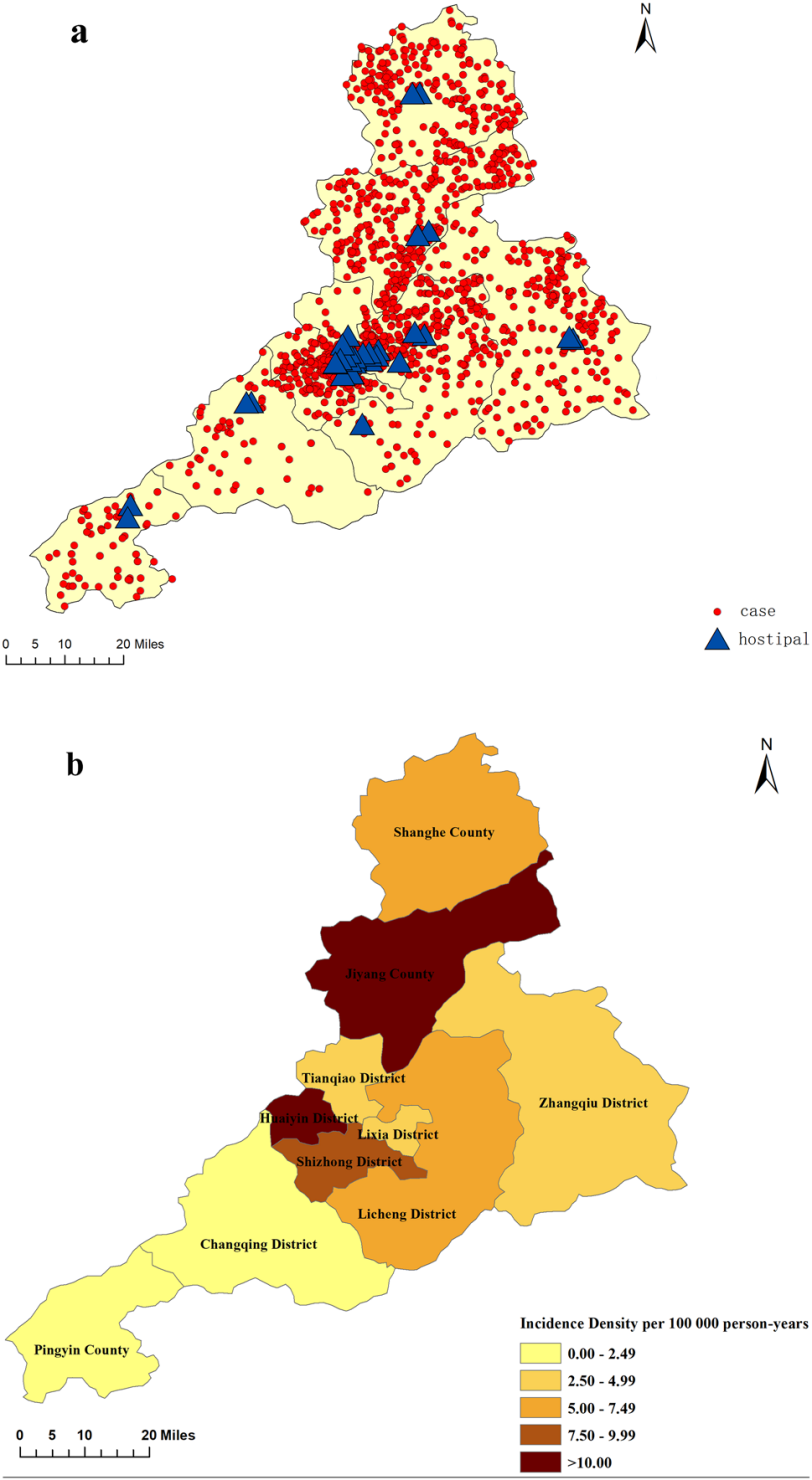
Spatial distribution of incidence density in Jinan from 2007 to 2021. All CO poisoning incidents (**a**), incidence density (**b**) in Jinan during 2007 to 2021.

**Figure 4:**
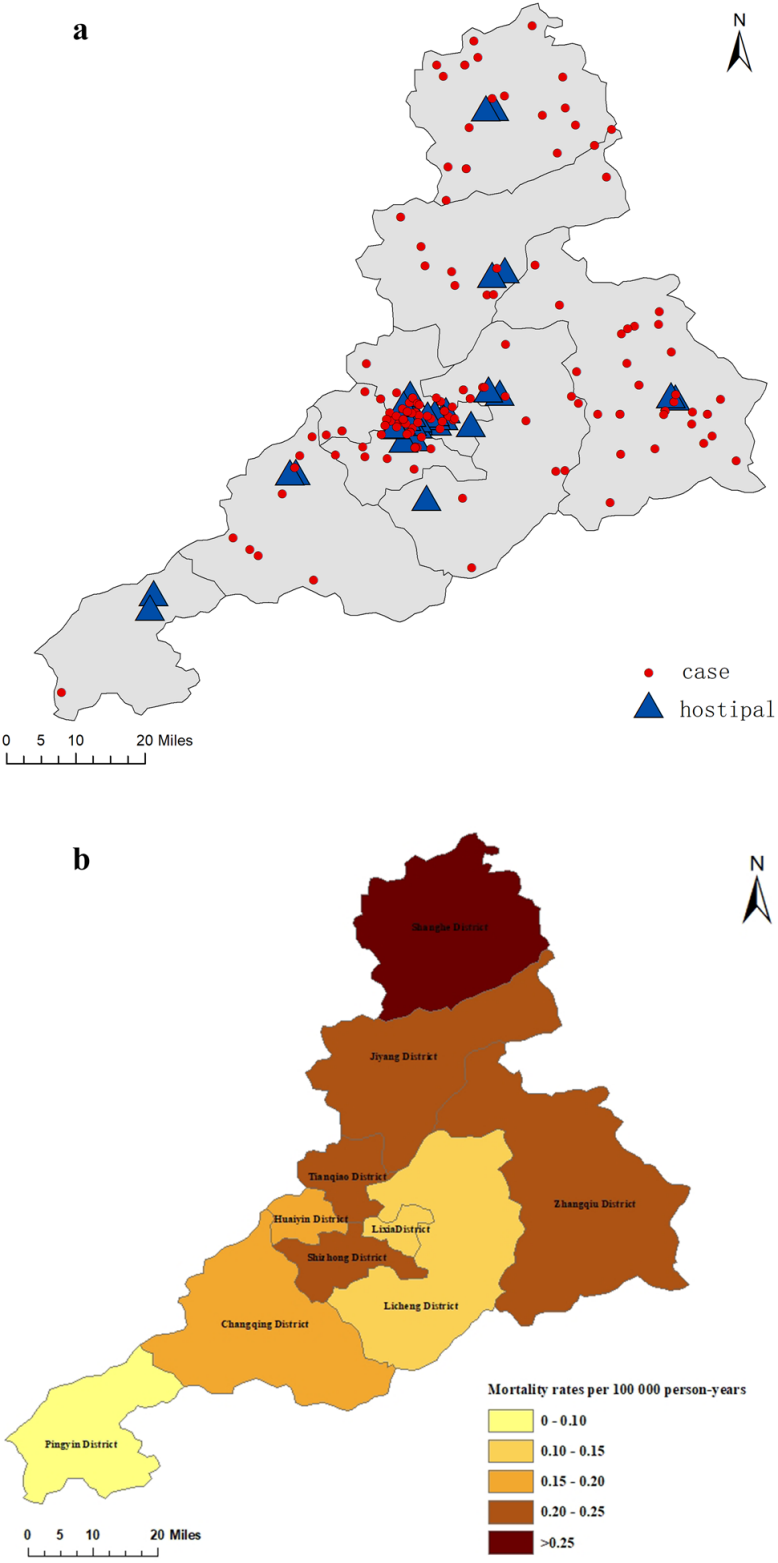
Spatial distribution of mortality rate in Jinan from 2007 to 2021. All CO poisoning deaths (**a**) and mortality rates (**b**) in Jinan during 2007 to 2021.

Spatial Distribution of Incidence Density across Administrative Districts in Jinan from 2007 to 2021was shown in Figs. 3 and 4. The incidence density of Jiyang and Huaiyin district ranked the highest among all districts, but in terms of mortality, Huaiyin district dropped to a moderate level, while Jiyang district remained at an upper-middle level. In addition, the incidence density in Shanghe, Zhangqiu and Changqing districts were lower in Jinan, but the mortality rate reached an upper-middle level.

### Temporal analysis

#### Year feature

**Figure 5:**
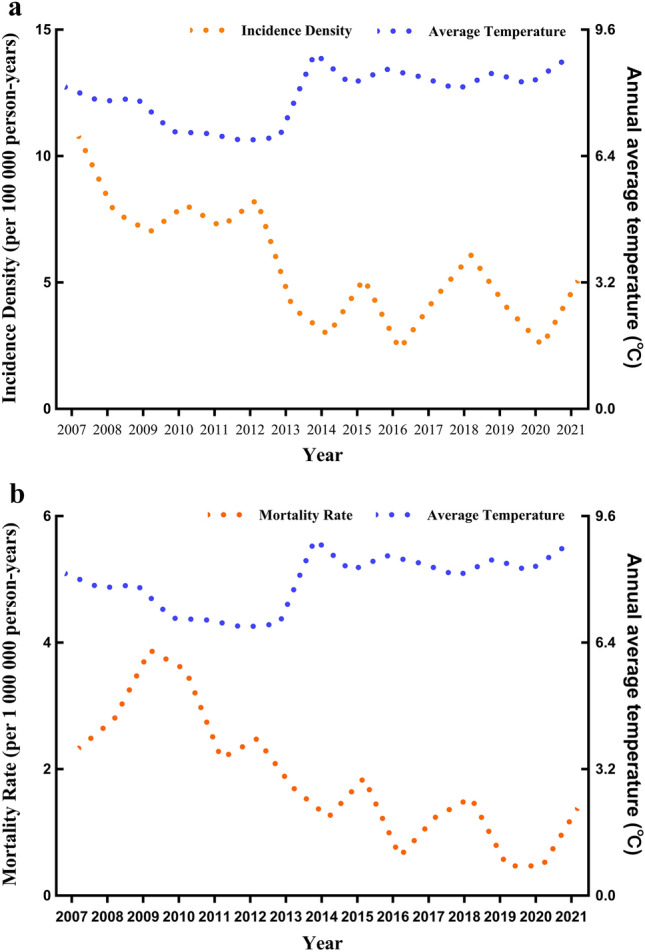
Incidence density (**a**) and mortality rate (**b**) with annual average temperature in Jinan from 2007 to 2021.

Epidemiological study shown variables attributed to CO poisoning such as time of year of exposure, time of day of exposure, socioeconomic status and temperature are reliable indicators which relate causes to outcomes of CO poisoning^21^. Figure 5 shown the overall downward trend of incidence density and mortality rate of CO poisoning in Jinan during the past 15 years, and the fastest decline was in 2013. However, in the overall downward trend, there are a few years with slight increases, such as 2012, 2015, 2018 and 2020. Especially, the inverse trend of CO poisoning incidence density and mortality rate was observed compared with the annual average temperature.

#### Month feature

**Figure 6:**
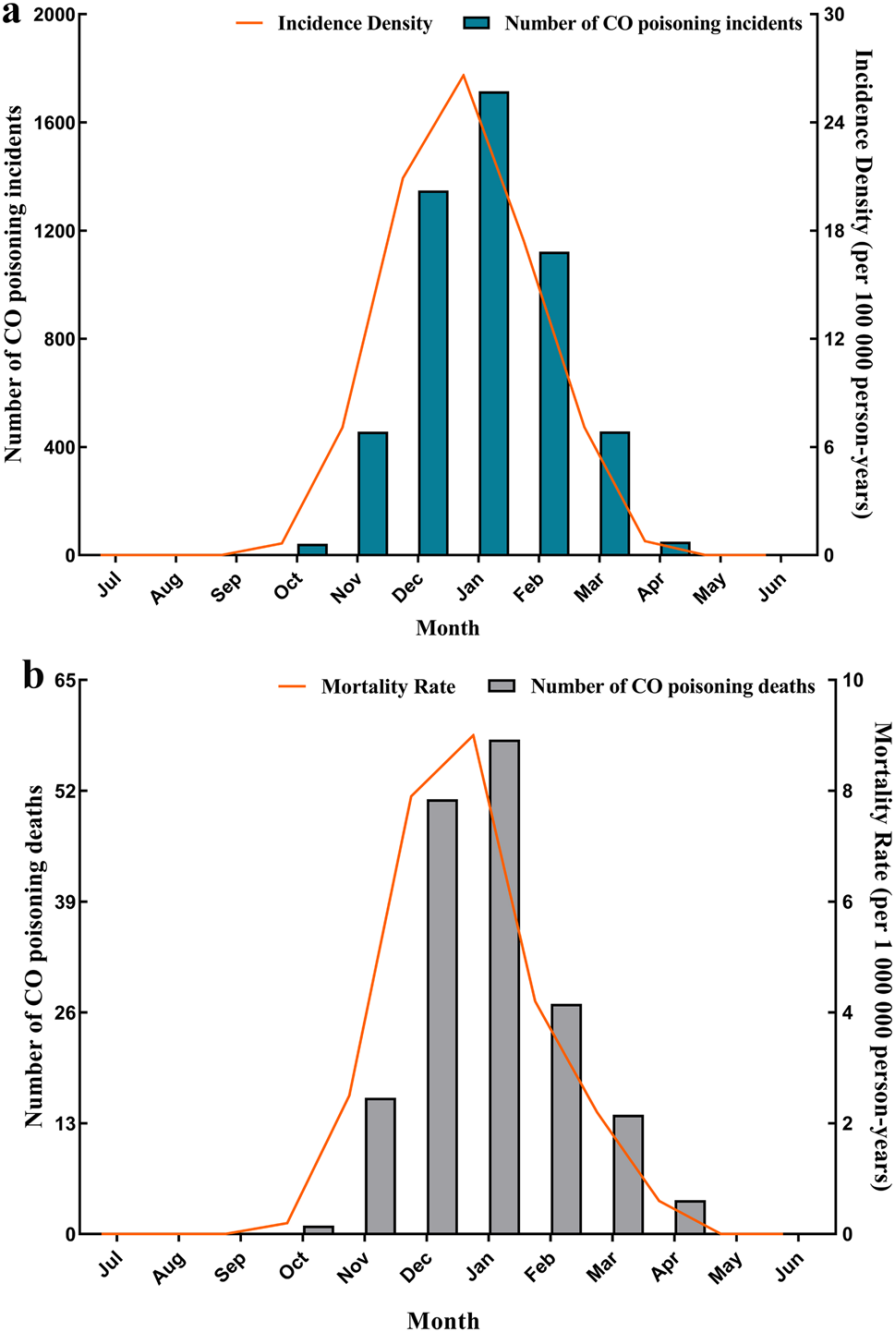
Incidence (**a**) and mortality (**b**) distribution for months in Jinan.

Figure 6 shown the monthly distribution of CO poisoning caused by household coal heating in Jinan from 2007 to 2017. It shown an obvious consistently seasonal characteristic of incidence and death with peaked month in January, and then decline rapidly.

#### 24-h feature

**Figure 7:**
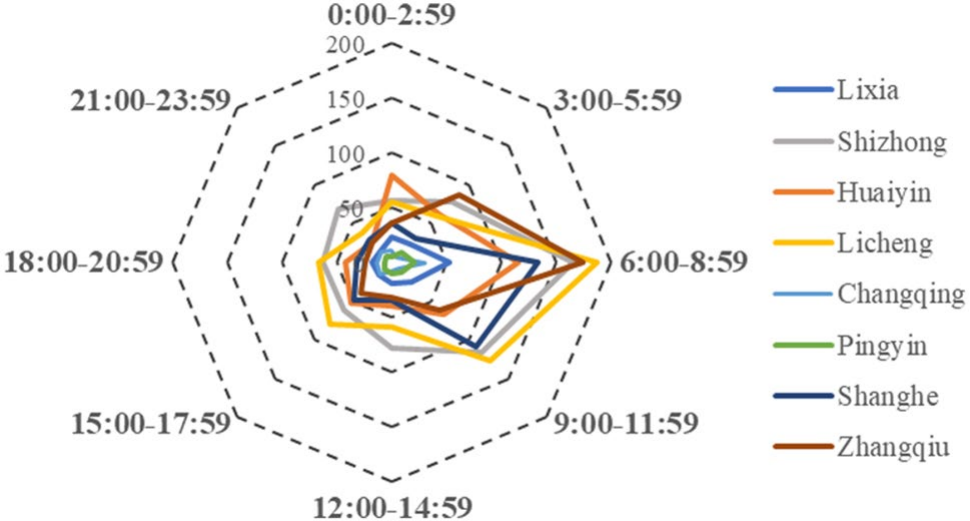
Incidence distribution at different times of day in Jinan.

As shown in Fig. 7, the occurrence of CO poisoning in Jinan had an obvious 24-h distribution characteristics. The highest of CO poisoning incidence density and mortality rates were between the hours of 6:00 am and 9:00 am, fewer districts were concentrated in 9:00 to 12:00. It is worth noting that this time reflected the time of visit, not the time of onset.

### Relationship of exposure, incidence and mortality

**Figure 8:**
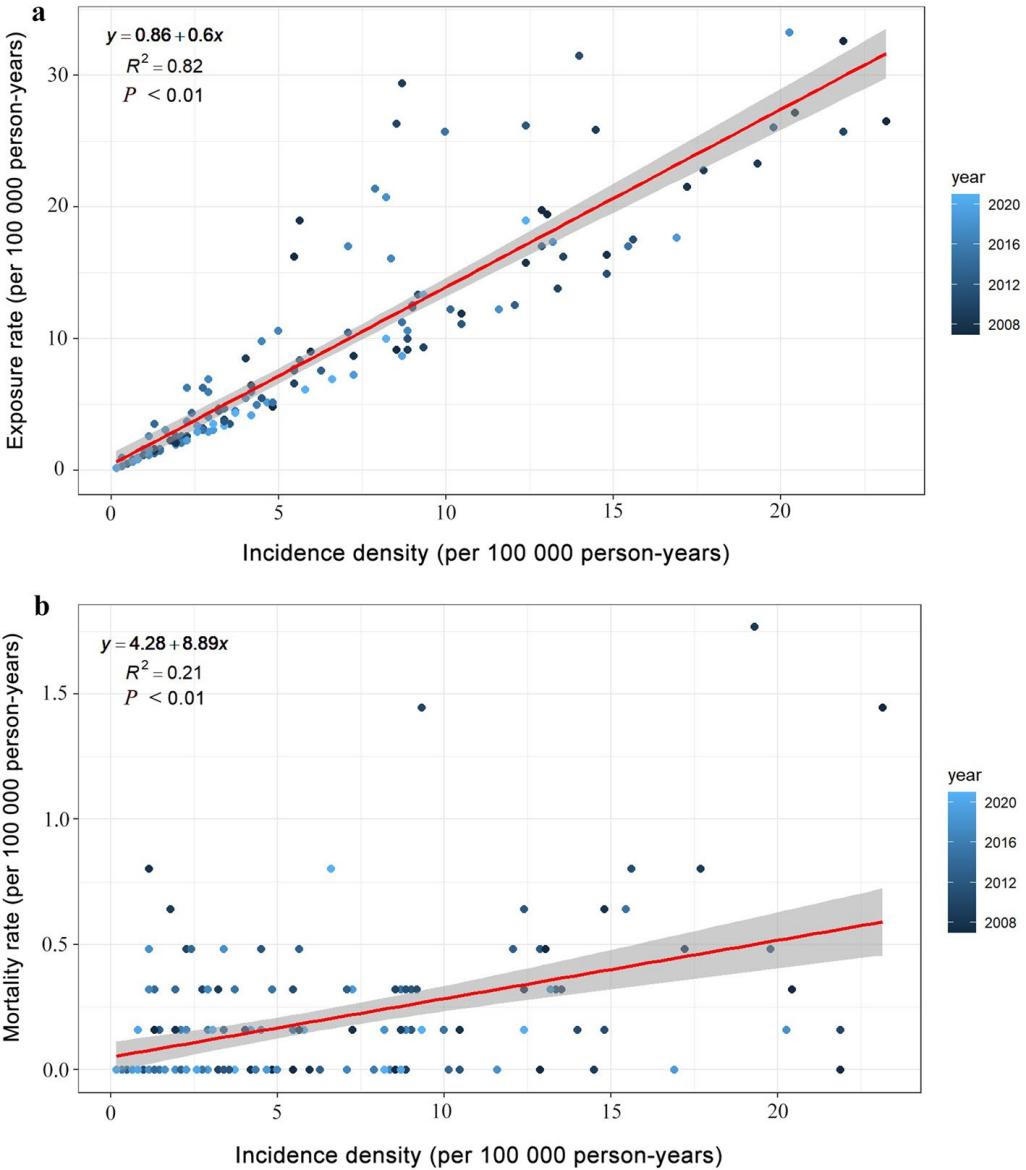
Relationship between exposure rate and incidence density (**a**), mortality rate and incidence density (**b**) of CO poisoning in Jinan from 2007 to 2021.

Exposure rate, incidence density and mortality rate were important indicators for evaluating CO poisoning development and medical rescue effect. In most cases mortality rates change as incidence density changes. Lower mortality rates relative to incidence density indicates faster diagnosis, better treatment available and effective health promotion; and higher mortality rates relative to incidence density indicates slower diagnosis, less treatment available and poor health promotion. We calculated the relationship between exposure rate, incidence density and mortality rate in Jinan from 2007 to 2021 (Fig. 8). Over time, CO poisoning is changing from high exposure (15.77 per 100,000 person-years) to low incidence (5.53 per 100,000 person-years) (*R*^2^ = 0.82, *P* < 0.01), and from high incidence (11.17 per 100,000 person-years) to low death (0.14 per 100,000 person-years) (*R*^2^ = 0.21, *P* < 0.01).

## Discussion

Our study has analyzed the current situation of household coal heating poisoning in a heavily incidence of CO poisoning city in China between 2007 and 2021, which provides an insight into how to reduce the occurrence of CO poisoning events. We found that over the 15 years, 5616 incidence cases of CO poisoning causes by household coal heating were reported, the cumulative incidence rate was 5.78 and the mortality rate was 0.19 per 100,000 person-years.

We observed a significant decreasing trend in 2013, and presented a mild fluctuation at this level in the following years. In 2013, the State Council of China issued the Air Pollution Prevention and Control Action Plan (APPCAP), which comprises ten specific tasks (Ten Tasks) with specific concentration targets to promote the use of new energy, adjust the energy structure and reduce the use of coal in order to reduce air pollution^22–24^. This measure has achieved significant results. In 2021, the area of central heating in Jinan was reached 304.58 million square meters, up 199.43% from 2013, the supply of natural gas was reached 1798.75 million cubic meters, up 228.68% from 2013, the coal to gas campaign has benefited 0.97 million households^25^. To this end, Jinan air pollution prevention and control actions have achieved additional indoor health benefits. In the following years, the occurrence of CO poisoning showed a state of up and down, it indicates the formulation of policy has an inhibitory effect on CO poisoning, but cannot play an absolute prevention. Therefore, the prevention of CO poisoning should be changed from government-led to "government + individual" two-pronged approach to awaken people's awareness of CO poisoning.

There was a seasonal pattern in the CO poisoning. In China, the temperature gradually turns colder from October, and winter is from November to February. The lowest temperature is in January, and the lowest temperature in Jinan can be below −10 ℃^26^. In our study, highest average daily number of CO related deaths occurred during the December to February, likely related to use of domestic heating with solid fuels. The results are similar to observations in the characteristics of CO poisoning in USA and Europe^27,28^. Almost everyone knows that heating in winter could cause CO poisoning, but this does not prevent the occurrence of CO poisoning^29^. In order to take more precise prevention and control measures, it may be possible to reduce the occurrence of CO poisoning by clarifying the time period when poisoning occur most frequently.

We used the radar chart to draw the time distribution of CO poisoning caused by coal heating in Jinan City within 24 h. In the past, people only focused on coal-heating poisoning often occurred in the cold season, but the period of onset was not further determined. In our study, we found that the occurrence of CO poisoning and the number of deaths began to increase from 0:00, and reached a peak between 6:00 and 9:00. The reason for this phenomenon may be that most people are in a deep sleep state before 6:00 in the morning, they have not noticed CO leakage. When they felt uncomfortable, rushed to the hospital, confirmed the diagnosis and the time usually between 6:00 and 9:00 in the morning. The main onset time of Huaiyin is from 0:00 to 3:00. The reason why Huaiyin is different from other areas may be because that Huaiyin has a large number of hospitals. It can be seen from Fig. 4 that the places with more cases in Huaiyin are closer to some hospitals. Therefore, most people could go to the hospital as soon as they perceive signs of discomfort, it causing the phenomenon that the number of incidences in Huaiyin was same as other areas, but the number of death distribution in Huaiyin is fewer. We can draw the following conclusions: (1) CO poisoning caused by coal heating mostly occurs during sleep, it reminded us that pay attention to the coal stove not too close to us when sleeping, and opening windows for ventilation. (2) Hospitals should be evenly distributed so that people in every region could receive effective treatment when they felt unwell.

Geographically, exposure and incidence rate reflected people's awareness of CO prevention and social health education level, mortality rate reflected medical treatment ability. In Jinan family CO poisoning study, we found that exposure and incidence rate of CO poisoning in urban areas is significantly higher than rural areas, the finding that is different with other studies^21,30^. The discrepancies with these studies might be explained by (1) the neglect of CO poisoning prevention in urban areas. Fewer residents use coal heating in urban areas of China, so that preventive measures in rural areas were more complete than urban areas, resulting in lower incidence in rural than urban areas; (2) Insufficient attention paid to CO poisoning by medical personnel in rural areas. The exposure and incidence rates of CO poisoning in some areas are almost same, indicating that some medical institutions only reported the people who got severe CO poisoning, did not report all the people who exposure CO poisoning, such as Pingyin and Changqing. To the above phenomena, we suggest that policy makers should enhance the attention of medical staff to CO poisoning, strengthen ideological training of CO poisoning in the whole society, policy implementation should not only focus on rural areas, but also in urban^31,32^. The death rate from CO poisoning in rural areas were higher than urban, which was consistent with other studies. It was because that medical resources in rural areas were relatively scarce, and the poisoned patients cannot receive effective treatment.

## Conclusions

Over the past 15 years, we have observed a decreasing trend of indoor CO poisoning, while it has still is an urgent indoor health problem in northern inland city of China. The problem of CO poisoning exposed in Jinan is that (1) the risk is worse in urban, (2) the onset time of high risk population is deep sleep period. An implication of China's air pollution control measures since 2013 may indirectly bring benefits to reduce indoor CO poison, but cannot play an absolute prevention. Therefore, we suggested popularize the central heating renovation, family health education and optimize the allocation of medical resources to further decrease the CO poisoning burden (Supplementary Information).

## Supplementary Information

Supplementary Information.

## Data Availability

The datasets used and/or analyzed during the current study are available from the corresponding author on reasonable request.
