# Supplementary material for: An urgent health problem of indoor air pollution: results from a 15-years carbon monoxide poisoning observed study in Jinan City
Source: Sci Rep. 2023 Jan 28;13:1619. doi: 10.1038/s41598-023-28683-0 (PMC9884191; doi:10.1038/s41598-023-28683-0)
Supplement: Supplementary file 1 — Supplementary Information. [file 41598_2023_28683_MOESM1_ESM.docx]

Supplementary tables 1. Numbers of carbon monoxide poisoning cases by districts during 2007 to 2021, Jinan City

| Districts | 2007 | 2008 | 2009 | 2010 | 2011 | 2012 | 2013 | 2014 | 2015 | 2016 | 2017 | 2018 | 2019 | 2020 | 2021 | Total |
| --- | --- | --- | --- | --- | --- | --- | --- | --- | --- | --- | --- | --- | --- | --- | --- | --- |
| Central city |  |  |  |  |  |  |  |  |  |  |  |  |  |  |  |  |
| Lixia District | 30 | 45 | 20 | 20 | 17 | 80 | 9 | 4 | 23 | 5 | 25 | 12 | 7 | 5 | 4 | 306 |
| Shizhong District | 127 | 92 | 110 | 136 | 92 | 56 | 21 | 23 | 26 | 18 | 52 | 51 | 29 | 17 | 51 | 901 |
| Huaiyin District | 136 | 37 | 90 | 84 | 57 | 56 | 13 | 34 | 54 | 14 | 28 | 3 | 18 | 26 | 23 | 673 |
| Tianqiao District | 80 | 26 | 25 | 17 | 12 | 28 | 13 | 8 | 9 | 13 | 18 | 21 | 19 | 16 | 58 | 363 |
| Licheng District | 144 | 65 | 8 | 34 | 34 | 123 | 39 | 17 | 62 | 49 | 44 | 126 | 105 | 17 | 77 | 944 |
| Rural region |  |  |  |  |  |  |  |  |  |  |  |  |  |  |  |  |
| Changqing District | 8 | 7 | 11 | 21 | 7 | 22 | 5 | 12 | 14 | 6 | 7 | 10 | 2 | 5 | 14 | 151 |
| Pingyin County | 2 | 12 | 3 | 14 | 6 | 5 | 3 | 5 | 17 | 2 | 7 | 7 | 4 | 5 | 21 | 113 |
| Jiyang County | 53 | 77 | 55 | 83 | 97 | 65 | 75 | 63 | 96 | 27 | 55 | 72 | 45 | 54 | 36 | 953 |
| Shanghe County | 34 | 35 | 54 | 53 | 87 | 77 | 35 | 15 | 30 | 18 | 14 | 7 | 16 | 16 | 19 | 510 |
| Zhangqiu District | 81 | 107 | 120 | 58 | 55 | 21 | 44 | 8 | 1 | 2 | 31 | 82 | 14 | 1 | 41 | 666 |
|  |  |  |  |  |  |  |  |  |  |  |  |  |  |  |  |  |
| Jinan | 695 | 503 | 496 | 520 | 464 | 533 | 257 | 189 | 332 | 154 | 281 | 391 | 259 | 162 | 344 | 5580 |

Supplementary tables 2. Administrative District Data of Carbon Monoxide (CO) Poisoning in Jinan from 2007-2021

| Districts | Number of Exposure | Number of Cases | Number of Deaths | Exposure Rate  (per 100,000 person-years) | Incidence Density  (per 100,000 person-years) | Mortality Rate  (per 100,000 person-years) |
| --- | --- | --- | --- | --- | --- | --- |
| Central city |  |  |  |  |  |  |
| Lixia | 380 | 306 | 11 | 3.85* | 3.1* | 0.11 |
| Shizhong | 1215 | 901 | 20 | 12.98* | 9.63* | 0.21 |
| Huaiyin | 988 | 673 | 10 | 15.69* | 10.69* | 0.16 |
| Tianqiao | 519 | 363 | 18 | 6.64* | 4.64* | 0.23 |
| Licheng | 1437 | 944 | 20 | 9.38* | 6.16* | 0.13 |
| Rural region |  |  |  |  |  |  |
| Changqing | 186 | 151 | 15 | 2.17* | 1.76* | 0.18* |
| Pingyin | 119 | 113 | 4 | 2.14* | 2.03* | 0.07* |
| Jiyang | 1046 | 953 | 20 | 11.54* | 10.51* | 0.22* |
| Shanghe | 1155 | 510 | 23 | 13.04* | 5.76* | 0.26* |
| Zhangqiu | 869 | 666 | 37 | 5.63* | 4.31* | 0.24* |

**P*＜0.05.

Supplementary figure 1


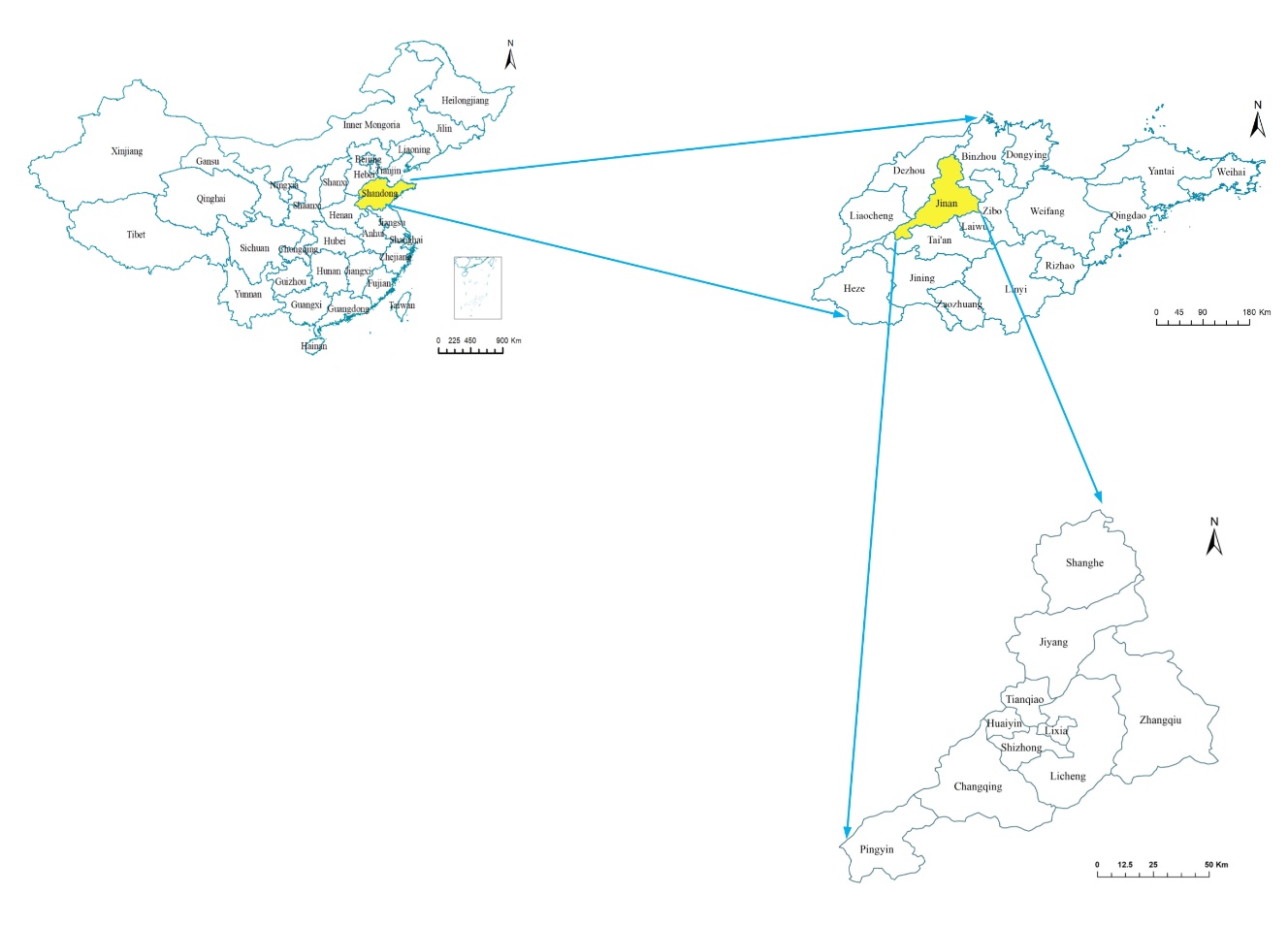


**Figure 1. Jinan map and location.**

Supplementary figure 2


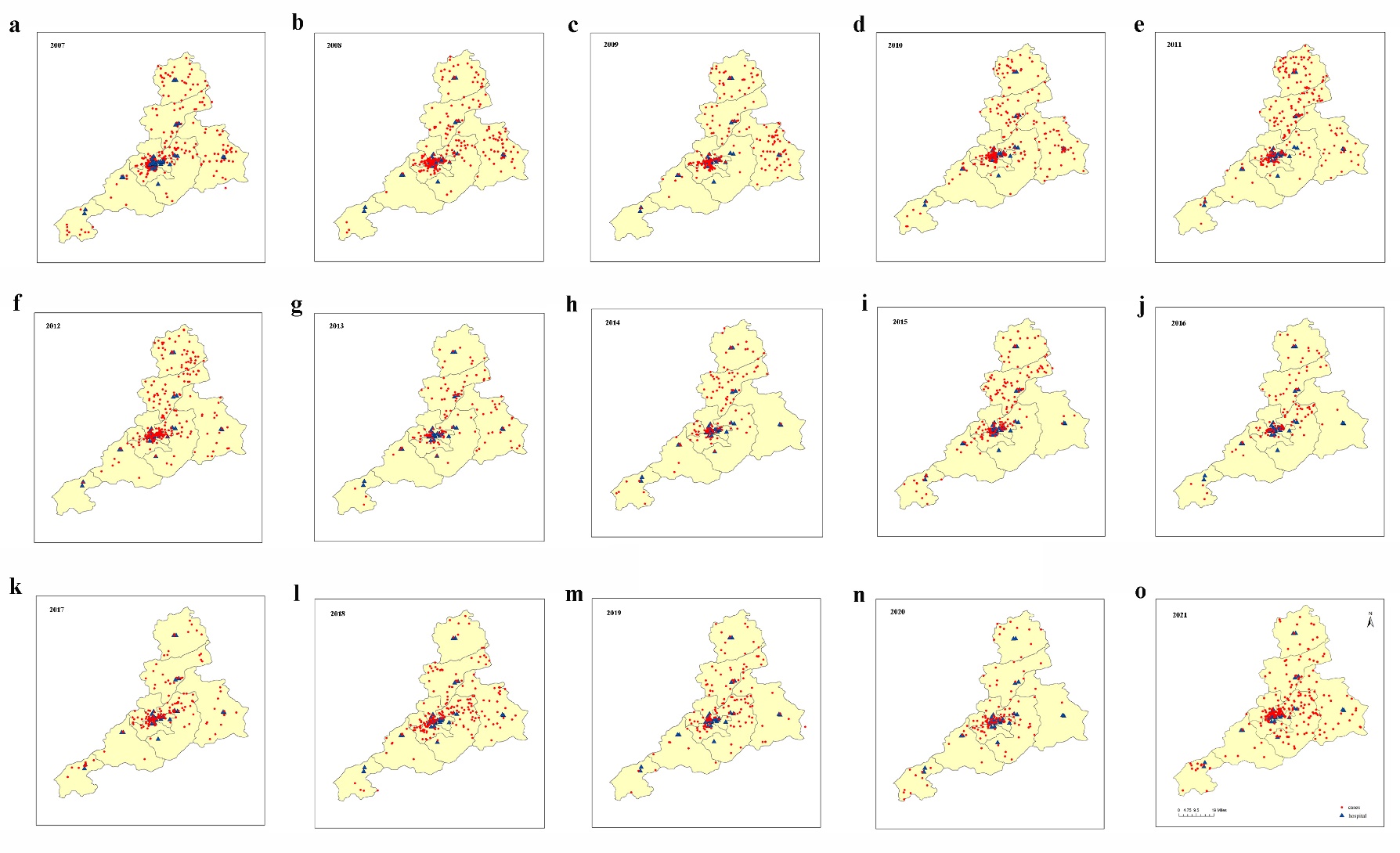


**Figure 2. Distribution of CO poisoning incidents in Jinan from 2007-2021.** CO poisoning incidents in Jinan from 2007 to 2021 (a-o). The red circles indicate CO poisoning cases, and the blue triangles indicate 34 hospitals in Jinan that are capable of CO poisoning treatment.

Supplementary figure 3


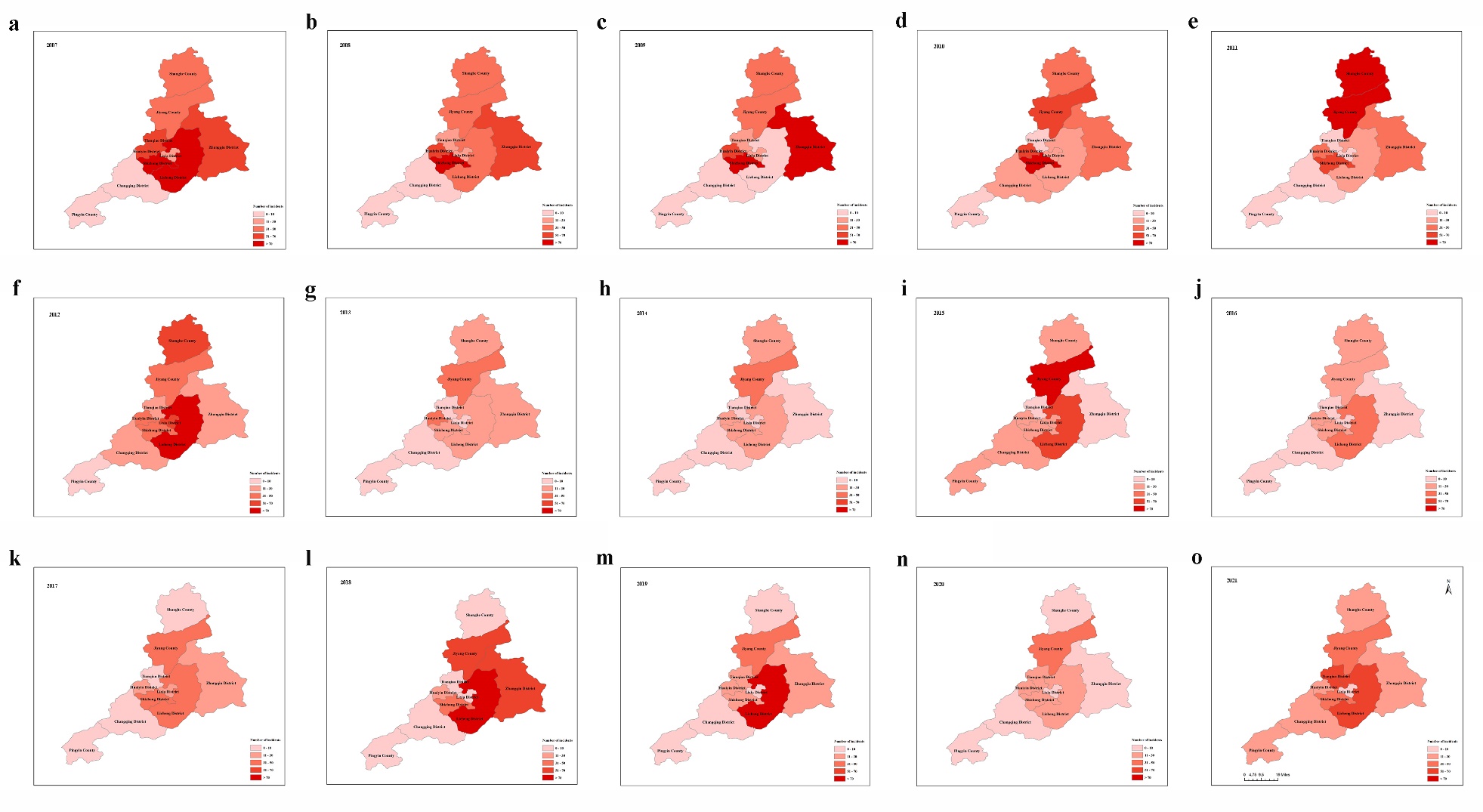


**Figure 3. The number of CO poisoning incidents in Jinan from 2007 to 2021.** CO poisoning incidents in Jinan from 2007 to 2021 (a-o), the darker the color, the more the occurrence.
